# Comparative Transcriptome Analysis in Tomato Fruit Reveals Genes, Pathways, and Processes Affected by the LEC1-LIKE4 Transcription Factor

**DOI:** 10.3390/ijms26146728

**Published:** 2025-07-14

**Authors:** Venetia Koidou, Dimitrios Valasiadis, Nestor Petrou, Christina Emmanouilidou, Zoe Hilioti

**Affiliations:** Institute of Applied Biosciences, Centre for Research and Technology Hellas, GR 57001 Thermi, Greece; vkoidoup@gmail.com (V.K.); dvalasiadis@certh.gr (D.V.); nestorpetrou@certh.gr (N.P.); xristinaemm@certh.gr (C.E.)

**Keywords:** tomato, fruit quality, fruit shape, L1L4, NF-Y transcription factor, RNA-sequencing, genome editing, ZFN

## Abstract

Tomato (*Solanum lycopersicum*) is a globally important crop, and enhancing its fruit quality and phenotypic traits is a key objective in modern breeding. This study investigates the role of the LEAFY-COTYLEDON1-LIKE4 (L1L4), an NF-YB subunit of the nuclear factor Y (NF-Y) transcription factor, in tomato fruit development using RNA-sequencing data from zinc-finger nuclease (ZFN)-targeted disruption lines. Differential gene expression (DEG) analyses of two independent *l1l4* mutant lines compared to the wild-type line revealed significant alterations in key metabolic pathways and regulatory networks that are implicated in fruit ripening. Specifically, *L1L4* disruption impacted the genes and pathways related to the fruit’s color development (carotenoid and flavonoids), texture (cell wall modification), flavor (sugar and volatile organic compound metabolism), and ripening-related hormone signaling. The analyses also revealed multiple differentially expressed histones, histone modifiers, and transcription factors (ERFs, MYBs, bHLHs, WRKYs, C2H2s, NACs, GRAS, MADs, and bZIPs), indicating that L1L4 participates in a complex regulatory network. These findings provide valuable insights into the role of L1L4 in orchestrating tomato fruit development and highlight it as a potential target for genetically improving the fruit quality.

## 1. Introduction

Fruit ripening is a complex developmental process that entails dramatic changes in color, texture, flavor, and aroma. These changes are tightly regulated by a network of transcription factors (TFs) that orchestrate the expression of the genes involved in various metabolic pathways [1]. Understanding the regulatory mechanisms underlying fruit ripening is crucial for developing strategies towards fruit quality improvement and shelf-life extension.

The NF-Y family TFs have been known to affect a vast range of biological processes (BP), from flowering to abiotic stress tolerance. In tomato, evidence suggests that they affect multiple fruit characteristics in terms of the quality, color, and flavonoid biosynthesis [2]. The NF-Y family comprises highly conserved proteins that modulate gene expression in a heterotrimeric fashion. The three subunits NF-YA, NF-YB, and NF-YC form a complex that binds to the CCAAT box in the promoter regions of genes [3], while the target gene specificity of the complex to target genes can also be synergistically influenced by other TFs, such as basic helix-loop-helix (bHLH) and basic leucine zipper (bZIP) [4,5]. The NF-Y family members are crucial master regulators of multiple downstream genes and BP, controlling agronomic traits that are important breeding targets. For instance, NF-YA8 coordinates the developmental aspects that can influence cotyledon growth, stem structure, inflorescence design, flowering timing, fruit size, and shape. Interestingly, the disruption of NF-YA8 had a substantial impact on a fruit’s morphology, its overall shape, dimensions, weight, sepal number, and fruit angle ratios [6].

In our previous studies, we employed a targeted gene disruption workflow on the NF-YB family member LEAFY COTYLEDON1-LIKE4 (L1L4 or NF-YB6), a known master regulator of fatty acid biosynthesis and seed storage proteins. Aside from its involvement in various aspects of plant development [7], expression in flowers, and unripe tomato fruits [8], we additionally investigated its influence on the metabolic profiles of tomato fruit [9]. Notably, the *l1l4* mutant lines showcased subtle differences in terms of the fruit size compared to the wild-type (WT) line, followed by significant changes in the fruit’s physiological parameters (e.g., moisture content, titratable acidity, etc.) and metabolic profiles (e.g., total polyphenols, antioxidants, oxalic and citric acid, fructose, and β-carotene). These altered metabolic profiles were also evident in seeds, showcasing a substantial variability in bioactive compounds compared to the WT seeds.

In this study, we further explored L1L4’s critical regulatory role as an NF-YB TF in the modulation of a wide network of genes, which can eventually affect various complex processes occurring in tomato fruit. For our analysis, we compared the fruit transcriptomes of two previously generated *l1l4* mutant lines (M7_5 and M29_2), both known for their distinct physiological and metabolic profiles compared to the WT, specifically regarding fruit fructose content, organic acid profiles, phenol content, and antioxidant activity. Our findings provide insights into the role of L1L4 in regulating fruit quality and metabolism in tomatoes, showcasing the vast effects of L1L4 in regulating downstream processes that involve a multitude of genes, epigenetic regulators, and TFs. Overall, this study aims to contribute valuable insights towards the development of elite tomato varieties.

## 2. Results

### 2.1. Fruit Morphometric Traits

**Figure 1:**
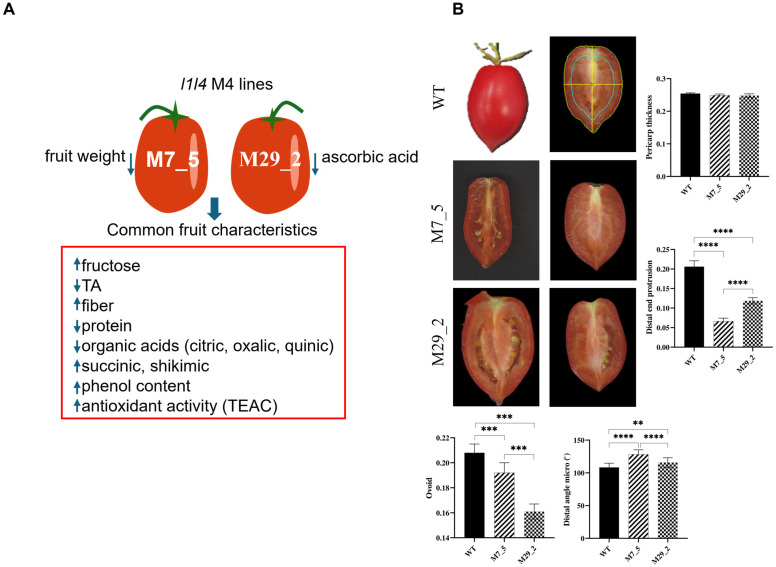
Morphometric traits of tomato fruits. (**A**) Impact of L1L4 disruption on fruit composition of the mutant lines M7_5 and M29_2 (based on [9]). TA: titratable acidity. Higher is shown by upward arrows, lower by downward arrows. (**B**) Morphometric variation in fruits from the wild-type (WT) line, and the two disruption lines M7_5 and M29_2 for distal end protrusion, distal angle micro, shape (ovoid), and pericarp thickness. Data are presented as means ± SD, *n* = 13–19, with the statistical significance determined by a one-way ANOVA. The differences among the groups were determined using Tukey’s post hoc test for multiple comparisons. The significance levels are indicated by asterisks as follows: ** *p* < 0.001, *** *p* < 0.0001, and **** *p* < 0.0001.

Figure 1A summarizes the compositional differences observed between the WT and the *l1l4* disruption lines (lines M7_5 and M29_2). The L1L4 disruption lines exhibited significantly lower moisture, protein, citric acid, oxalic acid, and quinic acid content compared to that of the WT. In contrast, both lines showed elevated levels of shikimic acid, and succinic acid was exclusively detected in the disruption lines, suggesting its accumulation. Furthermore, the disruption lines showed a significant increase in fructose content, while the glucose levels remained relatively stable. Line 5 also displayed a significantly higher fiber content, with line M7_5 showing an intermediate fiber level compared to the WT. Ascorbic acid was undetectable in line M29_2, while no significant differences were found between line M7_5, the WT, and M29_2 (where it was undetectable) (Figure S1).

This study aimed to identify the effects of *L1L4* gene disruption on tomato fruit morphology by comparing the fruit morphometric traits in mutant lines to the WT. Our results revealed significant differences in the fruit shape parameters between the WT and the two mutant lines (Figure 1B). The pericarp thickness did not present significant (*p* > 0.5) differences in the WT, M7_5, or M29_2 fruits, suggesting that L1L4 does not play a significant role in regulating pericarp thickness. However, the proximal angle of mutant line M29_2 was found to be significantly larger (177.17°) than that of the WT (133.4°) and mutant line M7_5 (178.88°). The distal angle of mutant line M7_5 was significantly larger (128.28°) than that of the WT (108.3°) and mutant line M29_2 (115.8°). The distal end protrusion of mutant line M7_5 was significantly smaller (0.066) than the WT (0.206) and mutant line M29_2 (0.119). Finally, the ovoid measurement was significantly lower in mutant line M29_2 (0.162) than in the WT (0.209) and mutant line M7_5 (0.192).

### 2.2. Differentially Expressed Genes Among Samples and Groups

**Figure 2:**
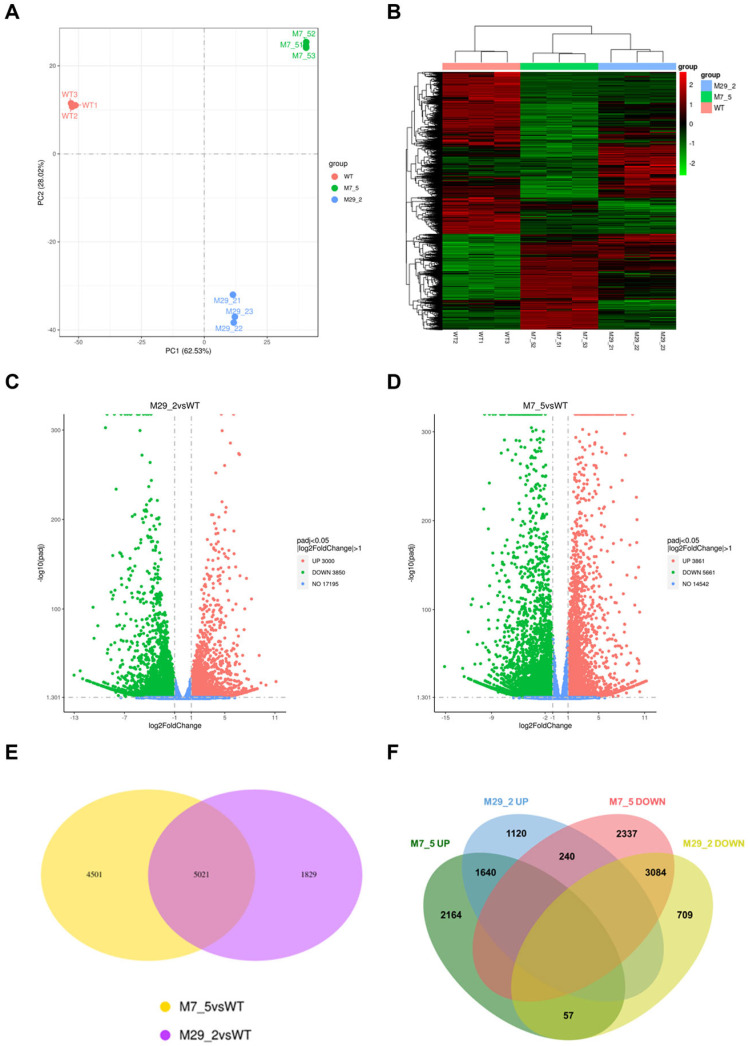
Gene expression differences between wild-type (WT) and *l1l4* mutant fruits. (**A**) Principal component analysis (PCA) was used to assess the variability between and within sample replicates of each group. (**B**) A heatmap visualizing the differentially expressed genes (DEGs) between the WT and *l1l4* mutant fruits, using thresholds of |log2(FoldChange)| ≥ 1 and an adjusted *p*-value (padj) ≤ 0.05. The red color indicates genes with high expression levels, and the green color indicates genes with low expression levels. (**C**,**D**) Volcano plots depicting the distribution of DEGs in the M7_5 vs. WT and M29_2 vs. WT comparisons. (**E**) The Venn diagram illustrates the overlap and unique DEGs for each mutant group (M7_5, M29_2) compared to the WT, highlighting 5021 common DEGs and 9522 and 6850 unique DEGs, respectively. (**F**) The Venn diagram illustrates common and unique upregulated or downregulated DEGs in the *l1l4* mutant fruits compared to the WT.

The fruit from the fourth generation of mutant lines M7_5 and M29_2 was chosen for RNA sequencing, while differential gene expression analysis was performed against the WT fruit (Heinz 1706). Principal component analysis (PCA) confirmed the inter-group variation and intra-group consistency, supporting the selection of these mutant lines for comparative analysis (Figure 2A). Gene expression analysis revealed distinct patterns across genotypes using hierarchical clustering. The heatmap (Figure 2B) displays differential gene expression, with the mutant lines (M7_5 and M29_2) forming separate clusters from the WT control. Notably, M7_5 exhibited a largely reversed expression profile compared to the WT, with genes highly expressed in the WT showing significantly reduced expression in M7_5, and vice versa. Volcano plots visualized the distribution of DEGs between mutants and the WT (Figure 2C,D). A Venn diagram (Figure 2E) highlighted the overlap (5021 genes) and unique gene expression profiles for each mutant group (M7_5: 9522 genes; M29_2: 6850 genes) relative to the WT. To identify the genes regulated by the ZFN-induced gene disruption, differential expression analysis was performed using DESeq2 (R package 1.20.0). Significant DEGs were defined as those with an FDR < 1 × 10^−3^, |log2FoldChange| ≥ 1, and an adjusted *p*-value ≤ 0.05, while excluding the genes with low expression levels (<50). Mapping short reads to the tomato reference genome allowed for the quantification of DEGs in each mutant compared to the WT, and between the mutant lines. Pairwise comparisons revealed the following: M7_5 vs. WT showed 9522 DEGs (2171 upregulated, 5661 downregulated) (Figure 2C); M29_2 vs. WT showed 6852 DEGs (3000 upregulated, 3850 downregulated) (Figure 2D); and M7_5 vs. M29_2 showed 5892 DEGs (2171 upregulated, 3721 downregulated). The Venn diagram (Figure 2E) illustrates the shared and unique differential gene expression (DEGs) between M7_5 vs. WT and M29_2 vs. WT (5021 shared DEGs). The greater number of DEGs in the M7_5 vs. WT comparison compared to M29_2 vs. WT suggests that the M7_5 mutation had a more pronounced impact on target gene expression. Conversely, the fewer DEGs in M29_2 vs. WT indicate a milder effect of the ZFN-targeted disruption in the M29_2 mutant.

Our data highlight L1L4’s critical role as a wide regulator of gene expression in tomato fruit, with a total of approximately 5000 shared DEGs between the mutant lines, and at least 3000 downregulated DEGs (Figure 2F), indicating a wider suppression than activation. In addition, both lines share 3084 downregulated DEGs and 1640 upregulated ones, indicating a particularly more suppressive role of L1L4 in the regulation of tomato fruit complex biological processes. On the other hand, M7_5’s higher number of both total and unique DEGs reveals that the induced L1L4 mutations in line M7_5 have a more prominent transcriptional impact, compared to line M29_2.

### 2.3. GO Term Analysis

**Figure 3:**
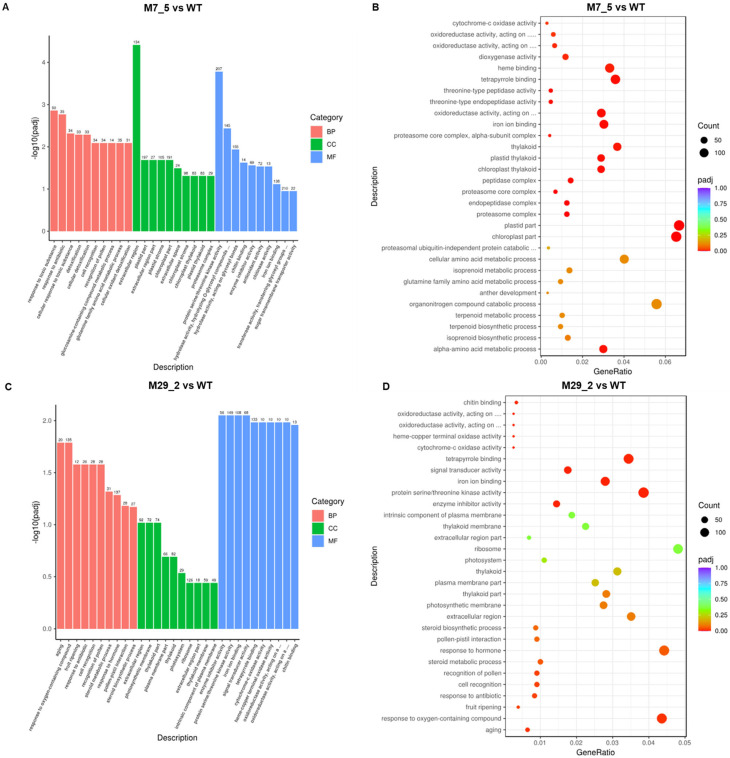
Enriched gene ontology (GO) analysis. (**A**,**C**) GO enrichment analysis histograms. The most upregulated and downregulated gene categories in the two mutant lines (A: M7_5, C: M29_2) are presented. The abscissa in the figure is the ratio of the number of differential genes linked with the GO term to the total number of differential genes, and the ordinate is the GO term’s level of significance of enrichment, expressed as -log10(padj). The different colors represent different functional categories: BP: biological processes; CC: cellular components; MF: molecular functions. (**B**,**D**) GO enrichment analysis scatter plots. The most significant 30 terms were selected for display. The size of each point represents the number of genes annotated to a specific GO term, and the color from red to purple represents the significant level of the enrichment; padj: adjusted *p*-value.

The enriched GO analysis results showed that the most upregulated and downregulated gene categories in the M7_5 mutant line included a response to toxic substances and antibiotics, cell recognition signals and glutamine amino acid metabolic processes (BP), an extracellular region, plastid and chloroplast expression (CC), protein serine/threonine kinase activity, hydrolase activity, transferase activity, and ion-ion binding processes (MF) (Figure 3A). The highest gene counts were detected in transferase activity, ion-ion binding, hydrolase activity, kinase activity, as well as extracellular region, chloroplast, and plastid part expression (Figure 3B).

In the M29_2 mutant line, the most upregulated and downregulated gene categories, according to the GO term analysis, include a response to oxygen-containing compounds and hormones (BP), an extracellular region, a photosynthetic region, a thylakoid part and ribosome expression (CC), protein serine/threonine kinase activity, and ion–ion as well as tetrapyrrole binding processes (MF) (Figure 3C). The highest gene counts were detected in ion–ion and tetrapyrrole binding, protein serine/threonine kinase activity, and ribosome, as well as a response to oxygen-containing compounds and to hormones (Figure 3D).

Taken together, both mutant lines display changes in kinase activity, indicating their shared responses in signaling and secretion, possibly influencing the fruit characteristics. A stress tolerance or resource reallocation response was more pronounced in M7_5, evidenced by the metabolic adjustments, such as amino acid metabolism and broad enzymatic activities. On the other hand, the M29_2 line had more pronounced regulatory processes, such as hormone signaling and photosynthetic efficiency, indicating a focus on energy production and an impact on photosynthetic machinery, which might be responsible for the changes in the fruit metabolic profiles and sugar content.

### 2.4. KEGG Enrichment Analysis

**Figure 4:**
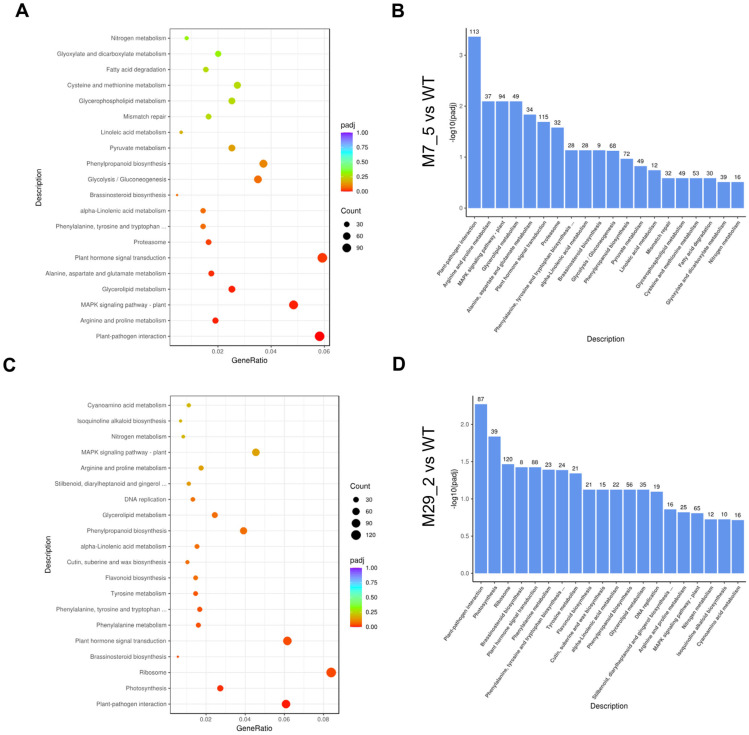
The Kyoto Encyclopedia of Genes and Genomes (KEGG) pathway enrichment analysis of the *l1l4* mutant fruits. KEGG pathway enrichment scatter plots comparing M7_5 vs. WT (**A**) and M29_2 vs. WT. (**C**). The *x*-axis represents the ratio of DEGs associated with the KEGG pathway to the total number of DEGs. The *y*-axis represents the KEGG pathway. The point size indicates the number of genes annotated to the KEGG pathway, while the color gradient (red to purple) indicates the enrichment significance level. KEGG pathway enrichment histograms comparing M7_5 vs. WT (**B**) and M29_2 vs. WT (**D**). The abscissa is the KEGG pathway, and the ordinate is the significance level of pathway enrichment.

The pathway enrichment analysis revealed metabolic or signal transduction pathways that were significantly over-represented, when comparing DEGs to the entire genome. In the M7_5 mutant fruits, the most significantly impacted gene categories involved the following: plant–pathogen interaction, plant hormone signal transduction, MAPK signaling, glycolysis/gluconeogenesis, glycerolipid metabolism, arginine and proline metabolism, and the proteasome pathway (Figure 4A). The plant hormone signal transduction, MAPK signaling, and plant–pathogen interaction pathways exhibited the highest number of gene annotations with 113 DEGs, followed by arginine and proline metabolism, as well as the MAPK signaling pathway and glycerolipid metabolism (Figure 4B). Accordingly, in the M29_2 mutant fruits, the most affected KEGG categories included ribosome, plant hormone signal transduction, MAPK signaling, plant–pathogen interaction, photosynthesis, brassinosteroid biosynthesis and the biosynthesis of phenylalanine, tyrosine and tryptophan (Figure 4C). The plant–pathogen interaction pathways had the highest number of associated genes (87), followed by MAPK signaling, plant hormone signal transduction, and ribosome (Figure 4D).

Interestingly, both mutant lines exhibited similar affected pathways, including the plant–pathogen interaction, MAPK signaling, and plant hormone signal transduction. However, the fruit of the M7_5 displayed a greater response on resource reallocation, as evidenced by the enriched glycerolipid metabolism, glycolysis/gluconeogenesis, arginine and proline metabolism, which possibly contributed to enhanced stress tolerance responses and modifications in the fruit metabolic profiles, such as the lipid and sugar contents. On the other hand, M29_2 appeared more enriched in the pathways related to photosynthesis, brassinosteroid biosynthesis, and ribosome activity, indicating a stronger need for energy capture, while M7_5 displayed a metabolic energy adaptation strategy.

### 2.5. Analysis of Histone-Related Genes

**Figure 5:**
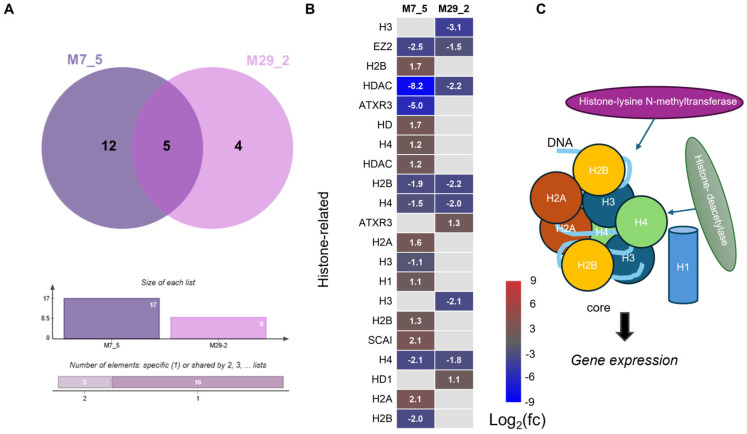
DEGs of histone and histone modifiers. (**A**) The Venn diagram displays common and unique histone-related DEGs in the M7_5 and M29_2 mutant lines compared to the WT. (**B**) The heatmap displays histone-related genes in the M7_5 and M29_2 mutant fruits, with red indicating upregulation and blu indicating downregulation compared to the WT. The values represent the fold change in gene expression. (**C**) The cartoon illustrates the affected histone-related genes in core histones (H2A, H2B, H3, and H4), linker histone H1, and histone modifiers (histone-lysine N-methyltransferase and histone deacetylase).

The analysis revealed significant differential expressions of the genes encoding histone modification enzymes and histone proteins in the *l1l4* disruption lines compared to the WT (Figure 5A, Table S4). The expression of several histone protein genes (*H2A*, *H2B*, *H3*, and *H4*) was altered in the mutant lines (Figure 5B). As can be seen in the heatmap, *Solyc06g075930.1*, *Solyc06g075800.1*, and Solyc11g066160.1 (encoding H2B and H4) were downregulated in both M7_5 and M29_2. However, Solyc11g010240.2, *Solyc03g071620.1*, *Solyc05g051515.1*, *Solyc06g074080.3*, *Solyc06g005430.1*, and *Solyc09g066100.3* (encoding histone H2A, H2B, H3, and H4) were upregulated specifically in the M7_5 line, while *Solyc07g052940.3* (Histone *H3*) was upregulated only in the M29_2 line. These changes in histone protein expression may influence the nucleosome assembly and chromatin structure. Both the M7_5 and M29_2 lines exhibited decreased expression of *Solyc03g044380.3* (*Histone-lysine N-methyltransferase*) (−2.45- and −1.45-fold change, respectively). The M7_5 line also showed a significant downregulation of *Solyc03g112690.1* (−5.00-fold change).

Conversely, *Solyc11g066840.2* (*Histone-lysine N-methyltransferase ATXR3*) was upregulated only in the M29_2 line (1.11-fold change). Downregulation of the *histone methyltransferases* (*HKMT*s) suggests a potential reduction in histone methylation, potentially affecting gene expression. Both the M7_5 and M29_2 lines exhibited decreased expression levels of *Solyc03g044380.3* (*Histone-lysine N-methyltransferase*) (−2.45- and −1.45-fold change, respectively). The M7_5 line also showed a significant downregulation of *Solyc03g112690.1* (−5.00-fold change). Overall, the two *l1l4* disruption lines displayed distinct expression profiles for histone-related genes. The unique expression patterns observed in each line likely reflect the specific impact of each L1L4 mutation on gene regulation. The differentially expressed histone-related genes encode core histones (H2A, H2B, H3, H4), linker histone H1, and histone modifiers such as histone-lysine N-methyltransferase and histone deacetylase (HDAC) (Figure 5C).

### 2.6. Analysis of TFs

**Figure 6:**
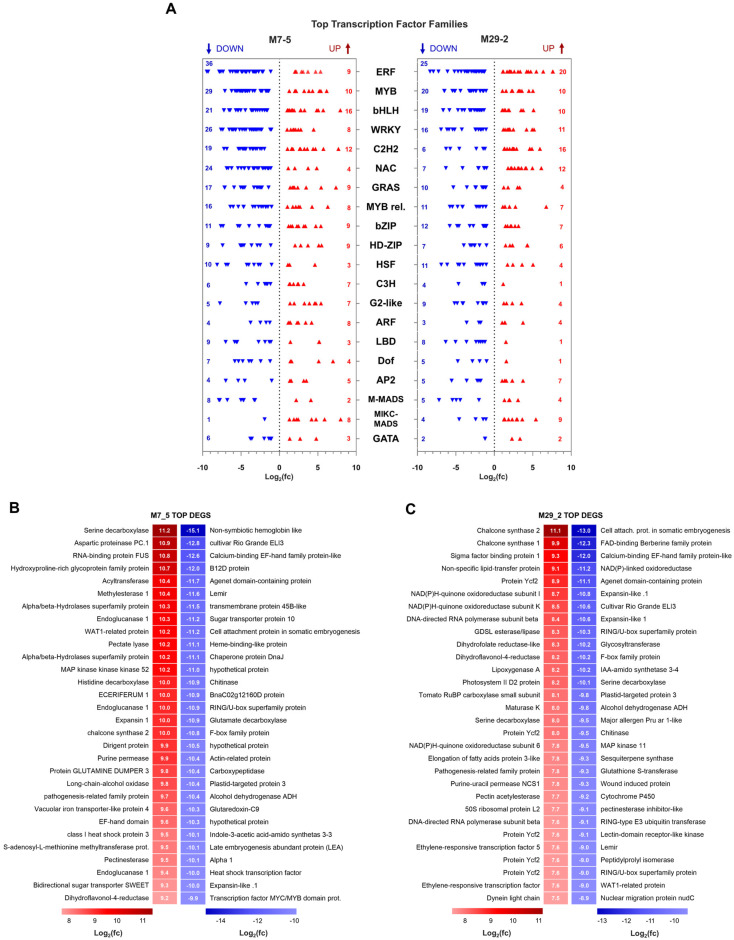
The top 20 families of the TFs and their members. (**A**) The heatmap displays the top DEGs in the M7_5 fruits, with red indicating upregulation and blue indicating downregulation relative to WT. The values represent the fold change in gene expression. (**B**) The heatmap displays the top DEGs in the M7_5 fruits. (**C**) The heatmap displays the top DEGs in the M29_2 fruits. Color scheme (red for upregulation, blue for downregulation) and representing values as a fold change compared to WT.

The implications of L1L4 disruption appear to significantly impact the network of transcriptional regulators and TFs, many of them known to affect fruit quality traits, including color, texture, flavor, and nutritional content. Detailed information regarding the number of DEGs across TF families in the mutant lines can be found in Supplementary Table S1 and Figure 6A. Interestingly, M7_5 displayed a greater number of TFs (543 DEGs) compared to M29_2 (402 DEGs), potentially due to differences in sensitivity to the mutations produced by the disruption of L1L4. The ERF family exhibited the highest number of DEGs, with an even number of 45 TFs in both mutants, followed by MYBs (44 DEGs in M7_5; 30 in M29_2), bHLHs (43 in M7_5; 29 in M29_2), WRKYs (38 in M7_5; 27 in M29_2), C2H2s (31 in M7_5; 22 in M29_2) and NACs (29 in M7_5; 19 in M29_2). In the case of the NF-Y family, both NF-YA and NF-YB revealed a more stable regulatory role of L1L4 in these subunits, evidenced by the consistent expression patterns in both lines. In contrast, NF-YC exhibited a variable response with two DEGs in M7_5 and none in M29_2, implying a line-specific affinity on this subunit. Overall, the magnitude of *L1L4* disruption impacted a vast network of TFs, highlighting the complex and crucial role of L1L4 in the gene expression landscape of tomato fruit.

The transcriptomics analysis of the M7_5 fruits revealed extensive DEGs compared to the WT. Focusing on the 30 most upregulated and 30 most downregulated genes (Figure 6B, Supplementary Table S2), we observed significant enrichment in the functional categories related to cell wall modification, stress response, and ethylene biosynthesis. Specifically, the upregulated genes included several involved in cell wall degradation, such as *Pectate Lyase* (10.21), multiple *Endoglucanases* (10.26, 9.99, 9.41), and *Pectinesterase* (9.45). The increased expression of these enzymes suggests a potential shift in cell wall dynamics, possibly reflecting a compensatory mechanism or an altered ripening process. Additionally, stress- and defense-related genes like *pathogenesis-related family protein* (9.71) and Class I *heat shock protein 3* (9.48) were also upregulated, potentially indicating a response to developmental cues or environmental challenges. The upregulation of *serine decarboxylase* (11.22) and *histidine decarboxylase* (*HD*) (10.02) points towards increased ethylene biosynthesis, a key regulator of ripening. Furthermore, the increased expression of *Chalcone synthase 2* (*CHS2*) (9.95) and *Dihydroflavonol-4-reductase* (9.24) suggests modifications to flavonoid metabolism, potentially influencing the fruit color and antioxidant capacity. Other noteworthy, upregulated genes implicate the involvement of other metabolic processes through genes such as *Aspartic proteinase PC.1* (10.94), *RNA-binding protein FUS* (10.81), *Acyltransferase* (10.35), *Methylesterase 1* (10.35), *MAP kinase kinase kinase 52* (10.18), and *Long-chain alcohol oxidase* (9.76). Conversely, several TFs, including *MYC/MYB* domain protein (-9.93) and *Heat shock TF* (−10.03), were downregulated, potentially disrupting the regulatory networks critical for fruit development and ripening. The downregulation of *late embryogenesis abundant protein* (*LEA*) (−10.11) suggests reduced stress tolerance. Furthermore, the decreased expression of *Indole-3-acetic acid-amido synthetase 3-3* (−10.13) and *Sugar transporter protein 10* (−11.24) suggests alterations in auxin and sugar metabolism, respectively. Additional downregulated genes, including *Glutaredoxin-C9* (−10.33), *Alcohol dehydrogenase ADH* (−10.36), *Glutamate decarboxylase* (−10.86), *Chitinase* (−10.91), *Heme-binding-like protein* (−11.14), *Cell attachment protein in somatic embryogenesis* (−11.16), and *non-symbiotic hemoglobin-like protein* (−15.12), likely contribute to the distinct ripening characteristics observed in the M7_5 fruits.

The top 30 upregulated genes in M29_2 are involved in a variety of BPs, including secondary metabolism, redox reactions, and transcriptional regulation (Figure 6C, Supplementary Table S2). Among the upregulated genes, two *CHS* genes (*Solyc05g053550.3* and *Solyc09g091510.3*) were identified, which are the key enzymes in the biosynthesis of flavonoids, a class of secondary metabolites with diverse biological activities. Additionally, several genes involved in redox reactions, such as *NAD(P)H-quinone oxidoreductase subunit I* (*Solyc11g021220.2*) and *dihydroflavonol-4-reductase* (*Solyc10g018140.2*), were also upregulated, suggesting a potential role of these genes in the response to oxidative stress. Furthermore, several TFs were found to be upregulated, including *ERF5* (*Solyc03g093550.1*) and a *lectin-domain receptor-like kinase* (*Solyc07g055690.2*), indicating a potential role of these genes in the regulation of downstream genes. On the other hand, the top 30 downregulated genes in M29_2 are involved in various cellular processes, including protein degradation, cell wall modification, and signaling transduction. Among the downregulated genes, a nuclear migration protein *nudC* (*Solyc06g051950.3*) was identified, which is involved in the regulation of nuclear movement during cell division. Additionally, several genes involved in protein degradation, such as a *pectinesterase inhibitor-like* (*Solyc06g005460.1*) and a RING-type *E3 ubiquitin transferase* (*Solyc06g074140.1*), were also downregulated, suggesting a potential role of these genes in the regulation of protein turnover. Furthermore, several genes involved in cell wall modification, such as a *pectin acetylesterase* (*Solyc03g025580.1*) and an *expansin-like* (*Solyc08g007090.2*), were found to be downregulated, indicating a potential role of these genes in the regulation of cell wall dynamics.

Interestingly, the transcriptomic analysis of the fruit from the *l1l4* mutant lines M7_5 and M29_2 revealed a set of DEGs with contrasting expression patterns. A significant number of genes were upregulated in line M7_5 (M7_5 UP) while they were simultaneously downregulated in line M29_2 (M29_2 DOWN) (Supplementary Table S3). These DEGs encompassed a diverse range of functions, including metabolism (e.g., alcohol dehydrogenase and hydroxysteroid dehydrogenase), stress response (e.g., dehydrin and late embryogenesis abundant proteins), protein processing (e.g., vacuolar-processing enzyme and cysteine proteinase), and transport (e.g., ABC transporter and sodium/hydrogen exchanger). Several genes encode proteins with roles in seed storage (e.g., 11S globulin, 2S albumin, and vicilin-like proteins), suggesting potential alterations in fruit nutritional composition. The presence of TFs like AP2-like ERF indicates disruptions in the hormonal signaling pathways, while changes in the genes related to DNA repair and cell wall modification further highlight the complex physiological changes occurring in these mutant fruit lines.

Similarly, there were 240 DEGs identified as downregulated in the M7_5 line but upregulated in the M29_2 line (Supplementary Table S3), while a subset of TFs and transcription regulators (TRs) was evident, reflecting potential regulatory divergence between these mutant genotypes. The analysis also revealed a total of 79 TF- and TR-related DEGs. The most represented family was the Pkinase group, with nine DEGs, followed by the C2 and C2H2 zinc-finger families, each with five members. The ERF, Pkinase_Tyr, and WRKY families were also notably represented, each contributing four contrasting DEGs. Additional TRs included TIR (3 DEGs), NAC (2 DEGs), and individual members of the NF-YA, bHLH, and MYB families (1 DEG each). This distribution highlights the involvement of diverse TF/TR families in the differential transcriptional response of the two *l1l4* mutant lines.

### 2.7. Identification of Fruit Metabolism and Quality Genes

**Figure 7:**
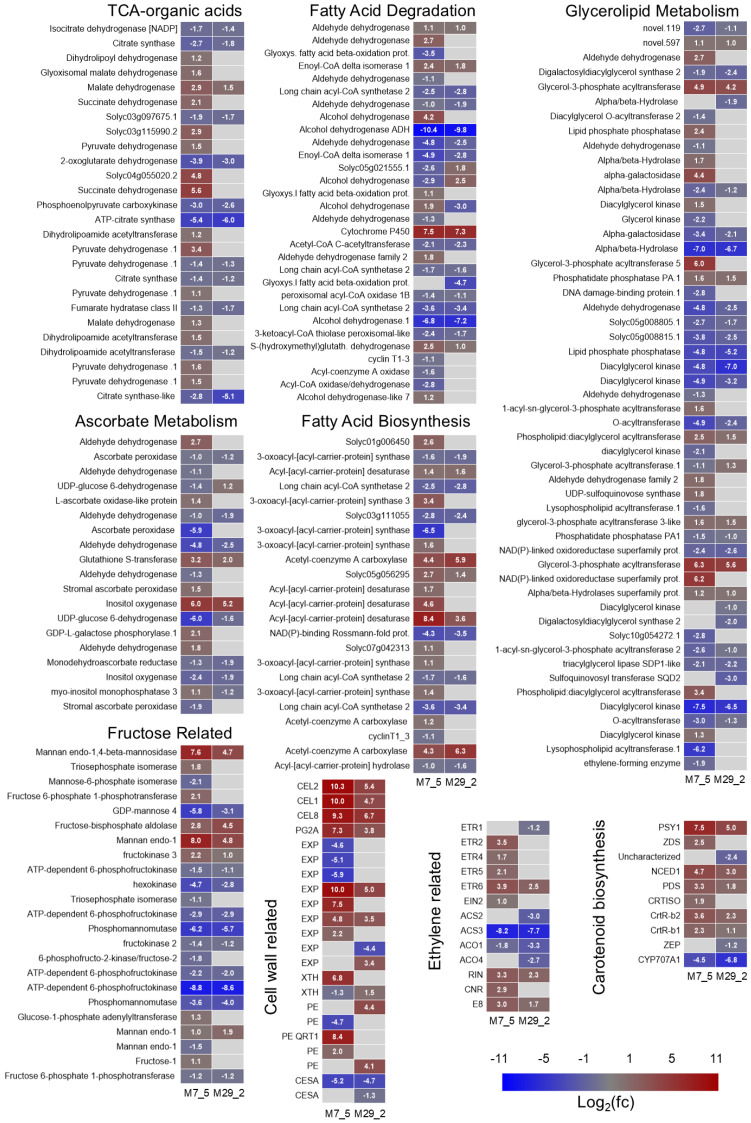
Differentially regulated tomato fruit metabolism and quality genes. Fruit quality genes, including fructose metabolism, the tricarboxylic acid (TCA) cycle, organic acids, cell wall modification, ethylene biosynthesis, carotenoid biosynthesis, ascorbate production, fatty acid biosynthesis and degradation, and glycerolipid metabolism. On the heatmap, the first line is dedicated to the M7_5 data, and the second line is for the M29_2 data. Red color indicates upregulation and blue indicates downregulation compared to the WT.

To understand the altered metabolic profiles of the mutant fruit lines and their impact on tomato fruit quality, we investigated the expression of key genes within several crucial metabolic pathways. These pathways included the following: fructose metabolism, the tricarboxylic acid (TCA) cycle, organic acid metabolism, cell wall modification, ethylene biosynthesis, carotenoid biosynthesis, ascorbate production, fatty acid metabolism, and glycerolipid metabolism (Figure 7, Supplementary Table S4). Our analysis revealed DEGs in each of these pathways, suggesting significant disruptions in the metabolic processes. Specifically, within fructose metabolism, genes encoding the fructokinase (FRK) isoforms exhibited differential regulation, potentially affecting fructose phosphorylation and utilization (Figure 7). Similarly, genes that were associated with the TCA cycle and organic acid metabolism, such as *citrate synthase* (*CS*), *aconitase*, and *malate dehydrogenase* (*MDH*), showed altered expression. Such changes could modify the levels of citric and malic acids, thereby impacting the fruit flavor. Alterations in cell wall texture were suggested by the DEGs encoding the cell wall modifying enzymes, such as polygalacturonase (PG), pectin methylesterase (PME), and cellulose synthase (CESA). The genes that were involved in ethylene biosynthesis, namely *1-aminocyclopropane-1-carboxylate synthase* (*ACS*) and *1-aminocyclopropane-1-carboxylate oxidase*, also showed differential regulation, potentially influencing the ripening process. Line M7_5 exhibited a complex pattern with upregulated ethylene receptors and downregulated ERF TFs, suggesting disrupted downstream signaling. Line M29_2 showed downregulated *ACS2*, potentially reducing ethylene biosynthesis. We further observed the altered expression of genes involved in carotenoid biosynthesis, particularly *phytoene synthase* (*PSY*), suggesting changes in the fruit color. Similarly, the genes that were involved in ascorbate biosynthesis, including *GDP-mannose pyrophosphorylase* (*GMP*), *GDP-L-galactose phosphorylase* (*GGP*), and *L-galactose-1-phosphate phosphatase* (*GPP*), were differentially regulated, potentially impacting the vitamin C content. Finally, the DEGs related to fatty acid and glycerolipid metabolism suggested alterations in the lipid composition, which could affect the fruit’s flavor, aroma, and cellular membrane integrity (Figure 7).

### 2.8. Validation of DEGs Genes with qRT-PCR Analysis

**Figure 8:**
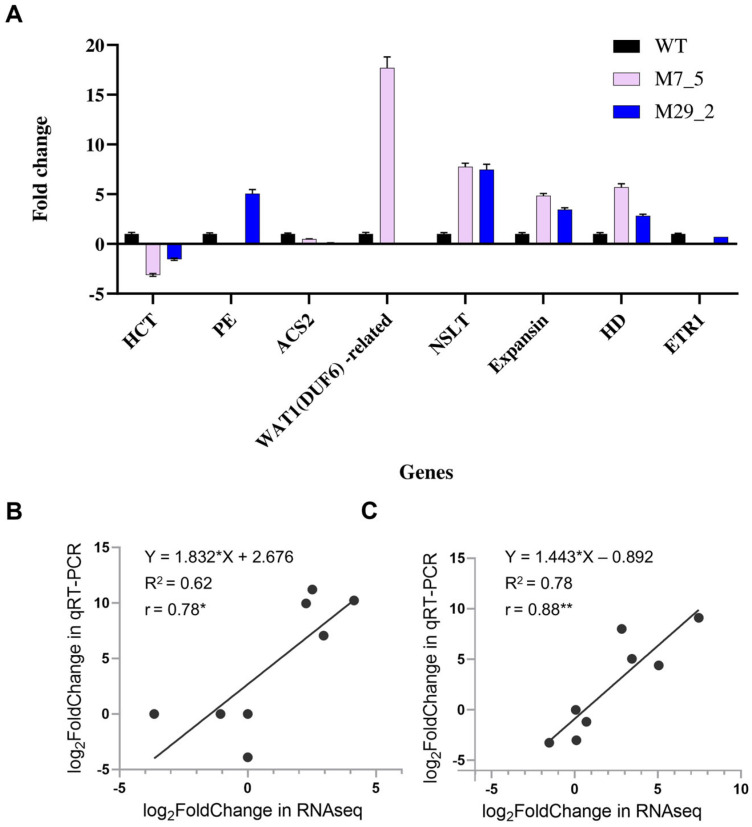
RT-qPCR validation of the DEGs and a quantitative assessment of the concordance between the RT-qPCR and transcriptomics data. (**A**) Fold change of mRNA expression of randomly selected genes *HCT1*, *PE*, *ACS2*, *WAT1(DUF6)*-related, *non-specific lipid transfer* (*NSLT*), *Expansin*, *histidine decarboxylase* (*HD*), *ETR1,* and *CHS2*. The genes were analyzed in fruit from two independent *l1l4* ZFN lines (M7_5 and M29_2) relative to the wild-type (WT) fruit. The expression levels were normalized to the reference gene *S. lycopersicum EF1-α* (*Elongation factor 1-α*, *Solyc06g005060*). Data are presented as the mean ± SD from three biological replicates (*n* = 3). Concordance between the two methods was assessed using Pearson’s correlation coefficient (r) and R^2^ values. (**B**) Analysis of the M7_5 samples revealed a significant positive correlation (r = 0.78, R^2^ = 0.62, *p* = 0.0202). (**C**) The M29_2 samples exhibited a more substantial and highly significant correlation (r = 0.88, R^2^ = 0.78, *p* < 0.01). Significance levels are indicated by asterisks as follows: * *p* < 0.05 and ** *p* < 0.001.

To validate the transcriptomics data, qRT-PCR was performed on nine randomly selected genes (Figure 8A). qRT-PCR is generally more sensitive than RNA-Seq, especially for genes with low expression levels. The selected genes are involved in various aspects of fruit metabolism, including cell wall modification, ethylene biosynthesis and signaling, flavonoid biosynthesis, phenylpropanoid metabolism, and ripening-related processes. Several genes exhibited strong agreement between the two methods. For example, *HCT* (*Solyc03g117600*), *CHS2* (*Solyc05g053550*), *non-specific lipid transfer* (*Solyc10g075070.2*), *Expansin* (*Solyc06g051800.3*), and *histidine decarboxylase* (*Solyc08g066250.3*) showed similar fold change directions and magnitudes in both the qRT-PCR and transcriptomics data, across both M7_5 and M29_2 lines (Figure 8A).

For assessment of the reliability and accuracy of the transcriptomic findings, the qPCR data were transformed to log2(2^−ΔΔCt^) values for a direct comparison with the log2-transformed transcriptomic fold changes. The concordance between the two methods was quantitatively assessed using Pearson’s correlation and R^2^ analysis (Figure 8B,C).

The quantitative assessment revealed high concordance between the two techniques across both mutant lines. In M7_5, a strong positive linear correlation (r = 0.78, *p* = 0.0202) was observed, with an R^2^ value of 0.62, indicating that the transcriptomics data explained 62% of the variance in the qPCR measurements (Figure 8B). An even higher degree of concordance was found in M29_2, with an exceptionally strong positive correlation (r = 0.88, *p* < 0.01) and an R^2^ of 0.78, demonstrating that the transcriptomics accounted for 78% of the variance in the qPCR results (Figure 8C). Beyond these quantitative correlations, individual gene expression patterns were also compared. Some genes exhibited agreement in only one of the two mutant lines, as they had contrasting expressions. For instance, *PE* (*Solyc03g083730*) provided data for the M29_2 line. The significant upregulation observed in M29_2 aligns well with the transcriptomics data. This suggests that *PE*, involved in cell wall modification, is significantly increased in the M29_2 mutant line. The qRT-PCR results for *ETR1* (*Solyc12g011330*) only provide data for the M29_2 line, where it shows a decrease in expression, aligning with the transcriptomics data. Similarly, *WAT1(DUF6)-related* (*Solyc11g012930.2*) aligned well in M7_5 but was low for M29_2 in the transcriptomics dataset. *ACS2* (*Solyc01g095080.3*) showed a significant discrepancy between the two methods. While the qPCR indicated minimal changes in expression in both conditions, the transcriptomics data suggested a strong downregulation in M29_2. In summary, the Pearson’s correlation coefficients (r > 0.7) and robust R^2^ values across both mutant samples affirm a strong alignment between qPCR and transcriptomics.

## 3. Discussion

Our earlier research revealed significant alterations in tomato fruit quality within the *l1l4* disruption lines. These changes encompassed a slight decrease in the fruit’s weight in line M7_5, as well as reduced titratable acidity, lowered moisture and protein contents, elevated fructose levels, modified organic acid composition, and an increased total phenol content coupled with enhanced antioxidant activity. These findings provided important insights into the role of L1L4 in regulating fruit quality and metabolic functions in tomatoes. The biochemical data, including shifts in organic acid profiles and sugar content, reinforce the idea that L1L4 acts as a key regulator of fruit metabolism.

Fruit morphology is essential in determining both the crop yield and consumer preference. Initial genetic studies identified several critical genes that markedly influence the fruit shape, such as *SUN*, *OVATE*, and *FAS* [10]. Extensive research on these genes has laid the groundwork for understanding the molecular mechanisms guiding fruit development. These genes produce proteins that collectively govern cell proliferation, cell elongation, and ultimately, the final shape of the fruit. Tomato lines M7_5 and M29_2 displayed altered fruit morphology compared to the WT, characterized by a diminished distal end protrusion and a more ovoid form. These morphological differences suggest that mutations in the *l1l4* lines affect the fruit shape. Supporting this conclusion, a disruption of the *NF-YA8* gene near its DNA-binding domain (DNA BD) has been reported to reduce fruit ovoidity by 40% in about half of the tomato plants [5]. In the M7_5 mutant line, the increased distal angle may result from modified patterns of cell division and expansion at the fruit apex. Previous research has established that the distal end of the tomato fruit is formed by a cluster of cells that divide and expand to create the fruit tip [11]. The larger distal angle observed in mutant line M7_5 implies alterations in cell division and expansion in this area, leading to a broader fruit apex. The reduced distal end protrusion and ovoid shape in both mutant lines may be attributable to the changes in cell wall composition and characteristics. Prior studies have demonstrated that the cell wall’s composition and properties influence the tomato fruit shape.

In the present study, RNA-sequencing analysis played a pivotal role in identifying specific genes and pathways regulated by L1L4. Notably, line M7_5 contains a 2-base pair deletion near the DNA BD of the L1L4 transcription factor, causing a premature stop codon [9]. In contrast, line M29_2 possesses a 1-base pair insertion within the coding sequence that shifts the reading frame. By comparing the transcriptomes of these mutant lines to the WT, we sought to pinpoint the genes and pathways controlled by L1L4 that contribute to the observed changes in fruit morphology and quality. The hierarchical clustering analysis revealed a dramatic reversal of gene expression patterns in the M7_5 mutant compared to the WT. This finding strongly suggests that the disrupted *L1L4* TF in M7_5 plays a crucial role in regulating gene expression, acting as a molecular switch. The observation that this TF acts as a molecular switch has broader implications for understanding gene regulatory networks. It suggests that TFs can act as crucial control points, and their disruption can have profound effects on the overall expression landscape. In the WT, the TF likely acts as either an activator or a repressor for specific sets of genes. In M7_5, the disruption of this TF gene leads to the derepression (activation) of genes it normally represses, while also causing the repression (decreased expression) of genes it normally activates. This reversal of gene expression patterns highlights the importance of disrupted TF in maintaining the proper balance of gene expression. Its disruption resulted in a global shift in the expression landscape, suggesting it acts upstream of numerous other regulatory factors. Further analyses, such as differential expression analysis and GO enrichment analysis, provided more insights into the specific genes and pathways affected by the TF disruption. Identifying the target genes of the TF is crucial to understanding its precise role in the cellular processes. The reversed gene expression patterns observed could lead to altered cellular functions and potentially contribute to a specific phenotype in the M7_5 mutant. Further phenotypic characterization of M7_5 is warranted to investigate the consequences of the gene expression reversal.

The transcriptomics analyses of fruit from the *l1l4* mutant lines M7_5 and M29_2 identified a collection of DEGs exhibiting opposing expression trends. A considerable number of genes were upregulated in line M7_5 (M7_5 UP) while they were being downregulated in line M29_2 (M29_2 DOWN). These DEGs spanned a wide array of functions, including metabolic processes (such as alcohol dehydrogenase and hydroxysteroid dehydrogenase), stress responses (including dehydrin and late embryogenesis abundant proteins), protein processing (like vacuolar-processing enzyme and cysteine proteinase), and transport mechanisms (such as ABC transporter and sodium/hydrogen exchanger). Several genes encoded seed storage proteins (for example, 11S globulin, 2S albumin, and vicilin-like proteins), indicating possible changes in the nutritional makeup of the fruit. The identification of transcription factors like AP2-like ethylene-responsive TF suggests disruptions in the hormonal signaling pathways. At the same time, alterations in the genes related to DNA repair and cell wall remodeling emphasize the complex physiological modifications occurring in these mutant fruits. The differing gene expression profiles between the *l1l4* mutant lines highlight the multifaceted role of the L1L4 complex in regulating fruit development. These gene expression differences likely contribute to the phenotypic variations observed during the development and ripening of the fruits from these two *l1l4* mutant lines.

Tomato fruit ripening involves significant epigenetic reprogramming [12,13,14], which is largely driven by the posttranslational histone modifications that influence the chromatin structure and gene expression [15,16]. The histone modification H3K27me3 has emerged as a key regulator of fruit ripening [17]. Despite this, direct genetic evidence linking histone methylases/demethylases to the regulation of flavonoid biosynthesis remains limited. However, a recent study observed increases in H3K27me3 levels at the *CHS1* locus in the peels of YB-CD-R fruit [5]. We discovered that differential expression of histone modification genes in the *l1l4* disruption lines provides evidence that the L1L4 complex influences epigenetic regulation in tomato fruit development. The downregulation of *HKMT*s and *histone deacetylases* (*HDAC*s) suggests that *L1L4* disruption may lead to a more open chromatin state, potentially increasing gene expression. Histone deacetylation is generally associated with gene repression. The strong downregulation of *Solyc03g112410.2* in both mutant lines, especially in M7_5, points to a potential disruption of gene-silencing mechanisms. However, the specific impact of these changes on gene expression and fruit development will depend on the target genes of these histone-modifying enzymes. In addition, the differential expression of the histone proteins (H1, H2A, H2B, H3, and H4) themselves is also significant. Changes in the histone protein levels can alter the stoichiometry of nucleosomes and impact the chromatin structure. The combination of altered histone modification and histone protein levels could lead to widespread changes in gene expression patterns.

Our analysis revealed a significant downregulation of various TF families, including WRKY, GRAS, NF-YB, Dof, bHLH, ARF, TCP, MYB, ERF, and C2H2, across both mutant lines. The altered expression patterns of these TFs provide insight into the disrupted regulatory networks governing fruit sets, cell division, differentiation, and ripening. WRKY TFs: WRKY TFs are involved in various plant processes, including stress responses, senescence, and hormone signaling [18]. Some WRKY TFs are also known to modulate fruit ripening processes, including controlling ethylene biosynthesis and regulating the expression of ripening-related genes [19]. The downregulation of WRKY TFs in the mutants suggests a disruption in these processes, potentially leading to alterations in fruit ripening. ERFs and TFs play key roles in regulating ethylene-responsive genes, which are involved in fruit ripening, senescence, and responses to various stresses [1]. The downregulation of ERFs in the M7_5 and M29_2 mutants suggests a potential disruption in ethylene signaling and its downstream effects on ripening-related processes. The MYB TF family is involved in diverse developmental processes and stress responses in plants, including fruit ripening. MYB TFs regulate the genes that are involved in anthocyanin biosynthesis, fruit softening, and other ripening-related pathways [20]. The observed downregulation of MYB TFs in the *l1l4* mutants suggests a potential alteration in these processes, possibly contributing to the observed ripening defects. During tomato fruit ripening, the accumulation of flavonoids on the fruit surface is governed by a transcriptional network. The bHLH family comprises transcription factors that are involved in regulating developmental processes, including fruit development and ripening [21]. Recent studies have shown a key role in the coordination with MYB proteins [22]. The distinct expression patterns of some TFs in the M7_5 and M29_2 mutants suggest that these genes may affect different signaling pathways or developmental stages during fruit ripening. The observed downregulation of numerous TFs in M7_5 and M29_2 tomato fruits suggests a disruption of the transcriptional regulatory networks that are essential for normal fruit development. The specific TF families affected provide clues regarding the processes that are impaired in these mutants.

The tomato is a widely consumed fruit and a model system for studying fleshy fruit development and ripening. The comprehensive RNA sequencing analyses of *l1l4* M7_5 and M29_2 tomato fruits revealed a complex network of DEGs that influenced various metabolic pathways critical for fruit quality, underscoring the significant impact of the induced mutations on gene expression in fruit tissue. The DEGs involved in fructose metabolism, TCA-organic acids, cell wall, ethylene biosynthesis, carotenoid biosynthesis, ascorbate biosynthesis, fatty acid metabolism, and glycerolipids suggest that these metabolic pathways are significantly affected in the mutant lines. The transition from a firm, green, unripe fruit to a soft, red, ripe fruit involves a cascade of biochemical and physiological changes. A central aspect of this process is the alteration of cell wall structure, which is primarily responsible for the fruit’s changing texture [23]. This modification is mediated by a suite of enzymes, including cellulases (EC 3.2.1.4), polygalacturonases (PGs; EC 3.2.1.15) [24], expansins [25], and cellulose synthases (CesAs; EC 2.4.1.21). These enzymes work in a coordinated manner to orchestrate the softening, texture development, and overall ripening process of the tomato fruit. Ethylene plays a pivotal role in regulating the expression of many of these enzymes [26]. It often acts as a trigger, initiating the ripening process and upregulating the expression of the genes encoding cellulases, PGs, and expansins. However, the regulation of CesAs by ethylene is less clear. Cellulases, polygalacturonases, expansins, and cellulose synthase represent a dynamic quartet of enzymes that play crucial and interconnected roles in tomato fruit ripening. While cellulases and PGs primarily degrade cell wall polysaccharides, expansins loosen the cell wall structure, and cellulose syntheses contribute to remodeling cellulose, maintaining or subtly altering the cell wall’s integrity. Understanding the interplay and regulation of these enzymes is essential for controlling fruit softening, texture development, and overall fruit quality. By manipulating the expression of the genes encoding these enzymes, breeders can develop tomato varieties with improved firmness, an extended shelf life, and tailored processing qualities.

In plants, ascorbate plays a crucial role in various physiological processes, including photosynthesis, cell growth, hormone signaling, enzyme cofactor activity, flowering, and stress responses [27,28]. It is a critical component of the ascorbate-glutathione cycle, which efficiently detoxifies the reactive oxygen species (ROS) generated during normal metabolic processes. Tomatoes are a significant source of ascorbate in the human diet, and understanding the metabolism in tomatoes can provide insights into improving their content in food crops. Ascorbate metabolism involves several enzymatic and non-enzymatic reactions, and the differential expression of genes encoding these enzymes can affect the ascorbate content in plants. The differential expression of the genes involved in ascorbate metabolism between the M7_5 and M29_2 tomato fruits suggests that these genes could contribute to their different ascorbate contents. The upregulation of *GDP-L-galactose phosphorylase* (*Solyc06g073320.3*) in M7_5 could increase the synthesis of ascorbate precursors, while the upregulation of *monodehydroascorbate reductase 1* (*Solyc09g009390.3*) could enhance the regeneration of ascorbate from its oxidized forms. On the other hand, the downregulation of *aldehyde dehydrogenase* (*Solyc06g060260.3* and *Solyc01g011510.3*) and *peroxidase* (*Solyc04g074640.3* and *Solyc11g018550.3*) in M7_5 could reduce the oxidative degradation of ascorbate and the consumption of its reduced form.

Disrupting *L1L4* resulted in tomato fruit with a significantly altered expression of genes involved in fatty acid biosynthesis and degradation. This may lead to decreased fatty acid activation and modification, potentially enhanced unsaturated fatty acid synthesis, and reduced β-oxidation. The latter could diminish energy production, alter precursor availability for volatile compound biosynthesis, and shift lipid metabolism. Upregulation of specific *ADH* genes and *Cytochrome P450 86A1* may represent compensatory mechanisms or shifts in metabolic flux, influencing volatile compound production and potentially modifying the fruit’s cuticular wax composition. The two *l1l4* disruption lines show varying gene expression patterns, which is likely due to the pleiotropic functions of *L1L4*. This finding is consistent with the established role of LEC1 and LEC1-like genes as critical regulators of fatty acid biosynthetic genes in *Arabidopsis* [29]. Similarly, in soybean, *GmLEC1* activates the genes involved in fatty acid biosynthesis while simultaneously repressing the transcription factors (e.g., GL2, MUM4, MYB5, and TT2) that negatively regulate lipid biosynthesis [30].

## 4. Materials and Methods

### 4.1. Plant Material

Tomato plants were cultivated in automated walk-in chambers that were located at the Institute of Applied Biosciences (Thessaloniki, Thermi, 57001, Greece). Intact, ripe tomatoes (Heinz 1706) were harvested from M4 progeny plants of two disruption lines and the WT. The mutant lines previously generated using ZFN genome editing technology and characterized for fruit composition and characteristics [9] included line M7_5 (referred to as line 3 in [9]), which has a 2-bp deletion. Line M29_2 (line 5 in [9]) has a 1-bp insertion in its coding sequence. These mutations were predicted to cause a premature stop codon in line M7_5 as well as nucleotide changes due to a shifted translation codon in line M29_2. For RNA sequencing analysis, the tomatoes were surface sterilized with 5% (*v*/*v*) commercial bleach for 10 min, followed by three rinses with deionized water. Fruits from the WT and the *l1l4* mutant lines (M7_5 and M29_2) were flash-frozen in liquid nitrogen and stored at −80 °C to preserve their integrity until RNA extraction. This method ensured that the fruits remained intact and maintained optimal quality for analysis. Each experiment consisted of three biological replicates, with each replicate representing a single fruit sampled at the red ripe stage from each genotype.

### 4.2. Digital Fruit Phenotyping

A morphometric analysis of longitudinally sectioned tomato fruits was performed using Tomato Analyzer software (version 3.0). WT and *l1l4* mutant fruits were scanned at 300 dpi and saved as TIF files before their analysis. The software quantified 13 to 19 fruits each from WT, M7_5, and M29_2 for distal end protrusion, fruit shape, distal angle, and pericarp thickness. To determine the effect of the mutation on fruit morphology, the percentage change in each parameter was calculated as (m − wt)/wt × 100%, where ‘m’ represents the mutant fruit value, and ‘wt’ represents the WT fruit value. Distal end protrusion was measured as the ratio of the area of the distal protrusion to the total area of the fruit, multiplied by 10. The fruit shape was measured as the ratio of fruit length to fruit width. The distal angle was measured as the angle between the best-fit lines drawn through the fruit perimeter on either side of the distal end point. The pericarp thickness was measured as the ratio of the average length of the pericarp along horizontal and vertical lines through the center of the weight to the average of the maximum height and maximum width.

### 4.3. RNA Extraction

Total RNA was extracted from the tomato samples using the Monarch Total RNA Miniprep Kit (NEB, Europe; T2010S) following the manufacturer’s protocol. DNase treatment was then performed to eliminate any residual DNA contamination. The RNA concentration was determined spectrophotometrically with a NanoDrop^TM^ 1000 (NanoDrop Technologies; Thermo Fisher Scientific, Inc., Wilmington, DE, USA). RNA integrity and quantity were further validated using the RNA Nano 6000 Assay Kit on a Bioanalyzer 2100 system (Agilent Technologies, Santa Clara, CA, USA).

### 4.4. Preparation of cDNA Libraries for Sequencing

An amount of 1 μg of RNA was used as input material for the sample preparations for sequencing. Sequencing libraries were generated using the NEBNext^®^ Ultra™ ΙΙ RNA Library Prep Kit for Illumina^®^ (NEB, Ipswich, MA, USA), adding individual barcoding code sequences to each sample. Afterwards, mRNA was purified using poly-T oligo-attached magnetic beads, followed by random fragmentation using the fragmentation buffer. The first-strand cDNA was then synthesized using a random hexamer primer and M-MuLV Reverse Transcriptase (RNase H-), and a second-strand cDNA synthesis was performed using DNA Polymerase I and RNase H. To complete the double-stranded cDNA, the remaining overhangs were converted into blunt ends via exonuclease/polymerase activities, and the NEB Next adaptor with a hairpin loop structure was used for hybridization. Following cDNA synthesis, cDNA fragments of preferentially 150~200 bp in length were purified with the AMPure XP system (A63880, Beckman Coulter, Beverly, MA, USA). PCR was performed using size-selected, adaptor-ligated cDNA and Phusion High-Fidelity DNA polymerase (M0530S, ΝΕΒ), and the resulting PCR products were purified using the AMPure XP system to assess their library quality on the Agilent Bioanalyzer 2100 system. The clustering of the index-coded samples was then performed on a cBot cluster generation system using the PE Cluster Kit cBot-HS (Illumina Inc., San Diego, CA, USA). Overall, nine cDNA libraries were constructed, three for each of the three genotypes. The nine generated cDNA libraries were sequenced on an Illumina Novaseq 6000 platform, in Novogene Co. (Cambridge, UK).

### 4.5. Bioinformatics Analysis

The raw paired-end reads were processed with in-house scripts to remove the adapter sequences, poly-N sequences, and low-quality reads. The trimmed reads were then mapped on the publicly available *S. lycopersicum* (tomato) genome (GCA_000188115.3) using the Hisat2 v2.1.0 short-read aligner, with the parameters, “–dta-cufflinks. FeatureCounts v1.5.0-p3 was used to count the reads mapped to each predicted gene, and the FPKM method was applied for the calculation of the gene expression levels. The raw read number was normalized to correct for the sequencing depth, and a statistical model was used to calculate the hypothesis test’s probability (*p*-value). Multiple hypothesis test corrections were applied to obtain False Discovery Rate (FDR) values. The DEGs were classified after a cut-off of an absolute value of log2 fold change ≥ 1 and an adjusted *p*-value ≤ 0.05. The assignment of the readings on a transcript level was performed using the htseq-count program, and the differential expression was performed using the DESeq2 R package (1.20.0). The fasta file of the predicted gene sets (version ITAG4) was downloaded from the FTP server of the Solanaceae Genomics Network (https://solgenomics.net/ftp/tomato_genome/annotation/, accessed on August 10, 2022). Enrichment analysis (clusterProfiler) was applied to identify significantly enriched biological functions or pathways. Gene Ontology (GO) term functional analysis was employed based on the Pfam terms, using the hmmscan tool and the Pfam-A.hmm database, while the GO terms were retrieved from the Pfam2GO. GO terms with a *p*-value of less than 0.05 were considered significantly enriched. In addition, KOBAS software 3.0 was employed to assign genes to their corresponding KEGG Orthology (KO) terms and conduct pathway enrichment analysis.

### 4.6. Real-Time Quantitative PCR (qRT-PCR) Analysis

A qPCR analysis was performed for nine genes. Briefly, total RNA was isolated from tomato tissues and extracted using the Monarch Total RNA Miniprep Kit (NEB, Europe), according to the manufacturer’s guidelines. The PrimeScript first-strand cDNA synthesis kit (TaKaRa Biomedicals, Otsu, Japan) was used to perform cDNA synthesis. The following primer pairs were used to amplify the target genes: *WAT1(DUF6)-related* (*Solyc11g012930.2*, F: AGGCCTAAGATGACACGCAG, R: CCAACCAACAATGCAAGCGA), *non-specific lipid transfer* (*Solyc10g075070.2*, F: AGGGAAAAGGCCCTCTAGGA, R: CGAGCAGTCAGTAAAAGGGC), *Expansin* (*Solyc06g051800.3*, F: TTACGGCGGAAGTGATGCTT, R: GGCATGGGATCCTGCGATAA), *ACS2* (*Solyc01g095080.3*, F: ATGGGATTTGAGATTGCAAAGACC, R: TTAACGAACTAATGGTGAGGG), *ETR1* (*Solyc12g011330*, F: ATGGGATCTCTTCTCCGG, R: CTAAGATTCAAGTACAACTCCATG), *CHS2* (*Solyc05g053550*, F: ATGGTCACCGTTGAGGAG, R: CTAAGTAGACACACTATGGAGC), *histidine decarboxylase* (*Solyc08g066250.3*, F: GCGATGCAGCATTATGTGGG, R: TACAACATGTGCCATGCCTCT), *HCT* (*Solyc03g117600*, F: CCCTCCTCCGTGCTCGTGA, R: CCCGGGTTAGTTTGAAGATTGACA) and *PE* (*Solyc03g083730*, F: TTCGAATTCAGTTTCCGCCG, R: GCTGTTTCGAGGCTAACGGA). *EF1-α* (*Solyc06g005060*; F: GCTGCTGTAACAAGATGGATGC, R: GGGGATTTTGTCAGGGTTGTAA) was used as a reference gene for normalization. Each reaction contained 1× buffer, 0.2 mM dNTPs, 0.2 mM forward and reverse primers for each gene, 1× EvaGreen^®^ dye (Biotium Inc., Hayward, CA, USA), 0.5U Kapa Taq DNA polymerase (Kapa Biosystems, Wilmington, MA, USA), and 20 ng of cDNA, in a 20 μL final reaction volume. The qPCR reactions were run in a Corbett Rotor Gene 6000 Thermocycler (Corbett Research, Sydney, Australia). The protocol included an initial denaturation step at 95 °C for 3 min, an amplification step with 35 cycles at 95 °C for 20 s, 57 °C for 20 s, 72 °C for 20 s, and a final elongation step at 72 °C for 7 min. Three biological replicates were prepared for each genotype, and three technical replicates were carried out for each biological sample. Each run included a non-template control reaction. The relative expression gene levels of the mutants compared to the WT samples were calculated using the 2^−∆∆CT^ [31] method. Gene expression data are expressed as the mean ± SE of the three biological replicates. qPCR data were plotted using GraphPad Prism version 9.0.

### 4.7. Statistical Analyses

Data analysis was performed using GraphPad Prism 6.0 (GraphPad, San Diego, CA, USA). Significant differences among group means (*p* < 0.05) were determined using ANOVA, followed by Tukey’s post hoc test for multiple comparisons to identify the specific differences between means. Transformation of the qPCR data using log2(2^−ΔΔCt^) was used to obtain the −ΔΔCt values for a direct comparison with the log2-transformed transcriptomic fold changes. Statistical analyses, using Pearson’s correlation and R^2^, provided a quantitative assessment of the concordance between the two methods.

## 5. Conclusions

This study investigated the role of the transcription factor L1L4 in tomato fruit using two ZFN-based disruption lines. The RNA-sequencing revealed that *L1L4* disruption significantly altered the gene expression that is related to plant–pathogen interactions, fruit metabolism, hormone signaling, and regulatory networks. The analysis of other related TFs, including the NF-Y family members, suggests a complex and coordinated regulatory network driving tomato fruit quality.

## Data Availability

Data supporting this study’s findings are available within the paper and its Supplementary Materials. Raw RNA-seq data has been deposited in BioProject under accession number PRJNA1289379.

## Supplementary Materials

The following supporting information can be downloaded at: https://www.mdpi.com/article/10.3390/ijms26146728/s1.

## Abbreviations

The following abbreviations are used in this manuscript:

ACS	1-aminocyclopropane-1-carboxylate synthase
ADH	alcohol dehydrogenase
BD	binding domain
BP	biological processes
CC	cellular components
CESA	cellulose synthase
CS	citrate synthase
DEG	differential gene expression
EF1-α	Elongation factor 1-α
ERFs	Ethylene Response Factors
FDR	False Discovery Rate
HD	histidine decarboxylase
HDACs	histone deacetylases
HKMT	Histone methyltransferase
FPKM	Fragments Per Kilobase of transcript sequence per Millions base pairs sequenced
FRK	fructokinase
GGP	GDP-L-galactose phosphorylase
GMP	GDP-mannose pyrophosphorylase
GPP	L-galactose-1-phosphate phosphatase
GO	Gene Ontology
KEGG	Kyoto Encyclopedia of Genes and Genomes
KO	KEGG Orthology
LEA	late embryogenesis abundant protein
L1L4	LEAFY-COTYLEDON1-LIKE4
MDH	malate dehydrogenase
MF	molecular functions
NF-Y	nuclear factor Y
NSLT	non-specific lipid transfer
padj	adjusted p-value
PCA	principal component analysis
PG	polygalacturonase
PME	pectin methylesterase
PSY	phytoene synthase
qRT-PCR	real-time quantitative PCR
SD	standard deviation
TCA	tricarboxylic acid
TF	transcription factor
WT	wild-type
ZFN	zinc-finger nuclease

## References

[1] Giovannoni J.J. (2004). Genetic regulation of fruit development and ripening. Plant Cell.

[2] Li S., Li K., Ju Z., Cao D., Fu D., Zhu H., Zhu B., Luo Y. (2016). Genome-Wide Analysis of Tomato NF-Y Factors and Their Role in Fruit Ripening. BMC Genom..

[3] Mantovani R. (1999). The Molecular Biology of the CCAAT-Binding Factor NF-Y. Gene.

[4] Myers Z.A., Holt B.F. (2018). NUCLEAR FACTOR-Y: Still Complex after All These Years?. Curr. Opin. Plant Biol..

[5] Wang J., Li G., Li C., Zhang C., Cui L., Ai G., Wang X., Zheng F., Zhang D., Zhang J. (2020). NF-Y plays essential roles in flavonoid biosynthesis by modulating histone modifications in tomato. New Phytol..

[6] Petrou N., Tsigarida N., Hilioti Z. Genome Editing of the NF-YA8 Gene Modifies Tomato Plant Architecture and Fruit Traits. Plants.

[7] Hilioti Z., Ganopoulos I., Ajith S., Bossis I., Tsaftaris A. (2016). A novel arrangement of zinc finger nuclease system for in vivo targeted genome engineering: The tomato LEC1-LIKE4 gene case. Plant Cell Rep..

[8] Hilioti Z., Ganopoulos I., Bossis I., Tsaftaris A. (2014). LEC1-LIKE paralog transcription factor: How to survive extinction and fit in NF-Y protein complex. Gene.

[9] Gago C., Drosou V., Paschalidis K., Guerreiro A., Miguel G., Antunes D., Hilioti Z. (2017). Targeted Gene Disruption Coupled with Metabolic Screen Approach to Uncover the LEAFY COTYLEDON1-LIKE4 (L1L4) Function in Tomato Fruit Metabolism. Plant Cell Rep..

[10] Rodríguez G.R., Muños S., Anderson C., Sim S.-C., Michel A., Causse M., Gardener B.B.M., Francis D., van der Knaap E. (2011). Distribution ofSUN, OVATE, LC, and FASin the Tomato Germplasm and the Relationship to Fruit Shape Diversity. Plant Physiol..

[11] Gillaspy G., Ben-David H., Gruissem W. (1993). Fruits: A Developmental Perspective. Plant Cell.

[12] Zhong S., Fei Z., Chen Y.-R., Zheng Y., Huang M., Vrebalov J., McQuinn R., Gapper N., Liu B., Xiang J. (2013). Single-Base Resolution Methylomes of Tomato Fruit Development Reveal Epigenome Modifications Associated with Ripening. Nat. Biotechnol..

[13] Lang Z., Wang Y., Tang K., Tang D., Datsenka T., Cheng J., Zhang Y., Handa A.K., Zhu J.-K. (2017). Critical Roles of DNA Demethylation in the Activation of Ripening-Induced Genes and Inhibition of Ripening-Repressed Genes in Tomato Fruit. Proc. Natl. Acad. Sci. USA.

[14] Tang Y., Liu X., Liu X., Li Y., Wu K., Hou X. (2017). Arabidopsis NF-YCs Mediate the Light-Controlled Hypocotyl Elongation via Modulating Histone Acetylation. Mol. Plant.

[15] Rea S., Eisenhaber F., O’Carroll D., Strahl B.D., Sun Z.-W., Schmid M., Opravil S., Mechtler K., Ponting C.P., Allis C.D. (2000). Regulation of Chromatin Structure by Site-Specific Histone H3 Methyltransferases. Nature.

[16] Karlić R., Chung H.-R., Lasserre J., Vlahoviček K., Vingron M. (2010). Histone Modification Levels Are Predictive for Gene Expression. Proc. Natl. Acad. Sci. USA.

[17] Lü P., Yu S., Zhu N., Chen Y.-R., Zhou B., Pan Y., Tzeng D., Fabi J.P., Argyris J., Garcia-Mas J. (2018). Genome Encode Analyses Reveal the Basis of Convergent Evolution of Fleshy Fruit Ripening. Nat. Plants.

[18] Eulgem T., Rushton P.J., Robatzek S., Somssich I.E. (2000). The WRKY superfamily of plant transcription factors. Trends Plant Sci..

[19] Wang L., Zhang X., Wang L., Tian Y., Jia N., Chen S., Shi N., Huang X., Zhou C., Yu Y. (2017). Regulation of Ethylene-Responsive SlWRKYs Involved in Color Change during Tomato Fruit Ripening. Sci. Rep..

[20] Allan A.C., Hellens R.P., Laing W.A. (2008). MYB Transcription Factors That Colour Our Fruit. Trends Plant Sci..

[21] Feller A., Machemer K., Braun E.L., Grotewold E. (2011). Evolutionary and Comparative Analysis of MYB and BHLH Plant Transcription Factors. Plant J..

[22] Loreti E., Betti F., Ladera-Carmona M.J., Fontana F., Novi G., Valeri M.C., Perata P. (2019). ARGONAUTE1 and ARGONAUTE4 Regulate Gene Expression and Hypoxia Tolerance. Plant Physiol..

[23] Brummell D.A. (2004). Cell Wall Metabolism during Maturation, Ripening and Senescence of Peach Fruit. J. Exp. Bot..

[24] Hadfield K.A., Bennett A.B. (1998). Polygalacturonases: Many Genes in Search of a Function. Plant Physiol..

[25] Cosgrove D.J. (2000). Loosening of Plant Cell Walls by Expansins. Nature.

[26] Osorio S., Alba R., Damasceno C.M.B., Lopez-Casado G., Lohse M., Zanor M.I., Tohge T., Usadel B., Rose J.K.C., Fei Z. (2011). Systems Biology of Tomato Fruit Development: Combined Transcript, Protein, and Metabolite Analysis of Tomato Transcription Factor (Nor, Rin) and Ethylene Receptor (Nr) Mutants Reveals Novel Regulatory Interactions. Plant Physiol..

[27] Smirnoff N., Wheeler G.L. (2000). Ascorbic Acid in Plants: Biosynthesis and Function. Crit. Rev. Plant Sci..

[28] Foyer C.H., Noctor G. (2011). Ascorbate and Glutathione: The Heart of the Redox Hub. Plant Physiol..

[29] Mu J., Tan H., Zheng Q., Fu F., Liang Y., Zhang J., Yang X., Wang T., Chong K., Wang X.-J. (2008). *LEAFY COTYLEDON1* Is a Key Regulator of Fatty Acid Biosynthesis in Arabidopsis. Plant Physiol..

[30] Manan S., Alabbosh K.F., Al-Andal A., Ahmad W., Khan K.A., Zhao J. (2023). Soybean LEAFY COTYLEDON 1: A Key Target for Genetic Enhancement of Oil Biosynthesis. Agronomy.

[31] Livak K.J., Schmittgen T.D. (2001). Analysis of Relative Gene Expression Data Using Real-Time Quantitative PCR and the 2^−ΔΔCT^ Method. Methods.

