# Supplementary material for: Comparative Transcriptome Analysis in Tomato Fruit Reveals Genes, Pathways, and Processes Affected by the LEC1-LIKE4 Transcription Factor
Source: Int J Mol Sci. 2025 Jul 14;26(14):6728. doi: 10.3390/ijms26146728 (PMC12295452; doi:10.3390/ijms26146728)
Supplement: Supplementary file 1 [file ijms-26-06728-s001.zip › Figure S1.pdf]

Trait	Wild-Type (WT)	M7_5/Line 3 (L1L4 Disruption)	M29_2/Line 5 (L1L4 Disruption)	Significance (vs. WT)
**Physical Characteristics**				
Fruit Size (Diameter/Length)	WT Value	No Difference	No Difference	NS
Fruit Weight (g)	WT Value	Lower (Not Significant)	Higher (Not Significant)	NS
**Color**				
Lightness (L*)	WT Value	Similar	Similar	NS
Yellowness (b*)	WT Value	Intermediate	Significantly Lower	Line 5: $p<0.05$
a*/b* Ratio	WT Value	Significantly Lower	Intermediate	Line 3: $p<0.05$
**Texture**				
Firmness	WT Value	No Difference	No Difference	NS
**Flavor & Nutritional**				
SSC	WT Value	Higher (Not Significant)	Higher (Not Significant)	NS
TA	WT Value	Significantly Lower	Significantly Lower	$p<0.05$
Taste Index	WT Value	Significantly Lower	Significantly Lower	$p<0.05$
Moisture Content	WT Value	Significantly Lower	Significantly Lower	$p<0.05$
Fiber Content	WT Value	Intermediate	Significantly Higher	Line 5: $p<0.05$
Protein Content	WT Value	Significantly Lower	Significantly Lower	$p<0.05$
Ash Content	WT Value	No Difference	No Difference	NS
Fructose Content	WT Value	Significantly Higher	Significantly Higher	$p<0.05$
Glucose Content	WT Value	Similar	Similar	NS
Ascorbic Acid	WT Value	Similar	Not Detected	Present in WT and line 3
Citric Acid	WT Value	Significantly Lower	Significantly Lower	$p<0.05$
Oxalic Acid	WT Value	Significantly Lower	Significantly Lower	$p<0.05$

Quinic Acid	WT Value	Significantly Lower	Significantly Lower	p<0.05
Shikimic Acid	WT Value	Significantly Higher	Significantly Higher	p<0.05
Succinic Acid	Not Detected	Detectable	Detectable	Present in Lines 3 & 5
**Phytochemicals & Antioxidants**				
Total Phenol Content	WT Value	Significantly Higher	Significantly Higher	p<0.05
Flavonoid Content	WT Value	Similar	Similar	NS
Lycopene Content	WT Value	Intermediate	Slightly Higher	NS
β-carotene Content	WT Value	Similar	Similar	NS
Antioxidant Activity (TEAC)	WT Value	Significantly Higher	Significantly Higher	p<0.05
Antioxidant Activity (ORAC)	WT Value	Significantly Higher	Significantly Higher	p<0.05

Figure S1: Comparison of Fruit Quality Traits in *L1L4* Gene Disruption Lines and Wild-Type Tomatoes. Data adapted from [9]. Note: "Significantly Higher" and "Significantly Lower" indicate $p < 0.05$ compared to WT as determined by statistical analysis. NS = Not Significant. " WT Value" indicates the value of the wild-type for comparison.
